# Evidence of self-organized criticality in time series by the horizontal visibility graph approach

**DOI:** 10.1038/s41598-022-20473-4

**Published:** 2022-10-07

**Authors:** Bardia Kaki, Nastaran Farhang, Hossein Safari

**Affiliations:** 1 Department of Physics, Faculty of Science, University of Zanjan, P.O. Box 45195-313, Zanjan, Iran; 2 Department of Physics, Isfahan University of Technology, P.O. Box 84156-83111, Isfahan, Iran

**Keywords:** Complex networks, Nonlinear phenomena, Phase transitions and critical phenomena, Statistical physics

## Abstract

Determination of self-organized criticality (SOC) is crucial in evaluating the dynamical behavior of a time series. Here, we apply the complex network approach to assess the SOC characteristics in synthesis and real-world data sets. For this purpose, we employ the horizontal visibility graph (HVG) method and construct the relevant networks for two numerical avalanche-based samples (i.e., sand-pile models), several financial markets, and a solar nano-flare emission model. These series are shown to have long-temporal correlations via the detrended fluctuation analysis. We compute the degree distribution, maximum eigenvalue, and average clustering coefficient of the constructed HVGs and compare them with the values obtained for random and chaotic processes. The results manifest a perceptible deviation between these parameters in random and SOC time series. We conclude that the mentioned HVG’s features can distinguish between SOC and random systems.

## Introduction

One of the most important parts of studying natural phenomena is classifying them into groups with the same characteristics. This could provide important information about forecasting capabilities and appropriate computational techniques required for further studies. Even though it seems straightforward at first sight, it is not, and it requires scientists to follow certain processes and strategies^1^. The problem becomes more intricate when there isn’t a comprehensive description of the subject system or the phenomenon encompasses a wide range of fields. Complex systems seem to confront both, because on the one hand there is no all-inclusive interpretation of these systems^2–4^, and on the other hand, they include everything from brain structure to insect colonies, price fluctuations in financial markets, condensate matter, Internet, Plasma and Solar physics, and even all human societies^3,5–10^. Given the breadth and intricacy mentioned, classifying complex systems is an outstanding issue that has attracted vast research interests^11–14^.

One of the most familiar aspects of complexity science is the nonlinear response of a system to small variations in its initial condition, usually referred to as chaotic behavior^15–17^. This behavior has been repeatedly reported in the literature across various fields including astronomy, biology, chemistry, economy, engineering, geology, etc.^18,19^. Considering the high sensitivity to initial conditions, identification of chaotic behavior in real-world data is more challenging than in artificial data sets^20^. Another aspect is the theory of SOC, in which a gradual energy supply leads the system to a critical state. Then, the system relaxes through a sequence (avalanche) of nonlinear energy-dissipative events that manifests a power-law-like behavior^5,21–25^. Here, “energy” corresponds to any interaction that could impose a phase transition from an original meta-stable state to another^26^, in contrast to the chaotic systems for which no stable state can be considered^27^.

Conventionally, a system with a slow driving rate and instant relaxations is most likely a SOC system if it could produce a wide range of avalanches with inverse dependence of frequencies on the event sizes. However, some variations from this interpretations have been discussed in the literature^28,29^. The chaotic and SOC systems along with significant differences exhibit some character resemblances and might even behave similarly^30^.

To this date, several algorithms have been developed to characterize complex systems^31,32^. One of the most popular techniques is the measure of Lyapunov’s exponent^33,34^, which determines how fast a very small distance between two originally close trajectories grow (or decrease) over time^35^. Another technique is to perform a cyclical analysis on time series (TS) and investigate whether the frequency spectrum exhibits a periodic pattern^36–38^. Furthermore, fractal dimension^39,40^, Kolmogorov-Sinai entropy^41^, and the network approach^42,43^ have been widely used in the identification and analysis of complex systems. The complex network theory could provide more convenient and manageable tools to conduct TS analysis^44–47^.

In the present study, we first validate the complexity of several observational and synthesis TS applying the detrended fluctuation analysis (DFA). Then, we construct the HVGs of these TS and calculate some of the graphs’ properties such as the degree distribution, maximum eigenvalue, and average clustering coefficient. Finally, we assess whether it is possible to distinguish between different categories of complex systems using these features. The remainder of this paper is organized as follows: in “DFA as a test for the complexity of a TS”, we explain the performed DFA. In “The classifier indicator”, we explain how the network theory could practically provide an indicator for random TS and discuss the possibility of determining random processes from other types (i.e., chaotic and SOC processes). In “HVGs of SOC systems” we first introduce several data sets and argue their complexity in applying the DFA. Then, we investigate the utility of the HVG approach in their identification. Finally, we present the conclusion in “Discussion”.

## DFA as a test for the complexity of a TS

A common point of most studies on complex systems is the extraction of patterns that govern the system’s dynamic and complexity testing is one of the first steps in such analyses^48^. Diagnosing the complexity of a system could be accomplished using a variety of methods mainly classified into three categories: nonlinear dynamic methods (based on the evaluation of the attractor properties in the phase space), the entropy-based methods (relevant to the disorder in a system) and fractality^49^. The fractal analysis gives an estimate of the complexity by measuring self-similarity (long-term persistence) in a TS. This analysis describes the global structures of a TS by means of a scaling coefficient $\alpha$, and a detrending estimator which corresponds to the fluctuations in the TS^50^. Hurst^51^ introduced the Hurst exponent as a scaling fractal analysis, and ever since, several methods have been developed to determine this exponent^52–55^.

Peng et al.^56^ established the DFA to determine the central trend and fluctuations of a TS via a polynomial fit. In this methodology, the time profile of a given data sample *x*(*i*) for $i=1, 2 .. N$ is defined:

1 $$\begin{aligned} y(j)=\sum ^{j}_{i=1}(x(i)-\bar{x}), \end{aligned}$$

where $\bar{x}$ is the average of the sample. The profile is divided into *l* equally spaced segments (blocks) with a total number of $N_{l}=(N/l)$ data points in each segment. The corresponding profile of each block is then fitted with a polynomial function which represents its local trend, $y_{l}(j)$. Subtracting the local trend from the original profile in each segment gives the characterization of the fluctuation:

2 $$\begin{aligned} F^{2}(l)=\frac{1}{l}\sum ^{l}_{j=1}{\bigg (y(j)-y_{l}(j)\bigg )}^{2}. \end{aligned}$$

Repetition of this procedure for various choices of *l* gives *F*(*l*) which follows $F(l)\propto l^{\alpha }$. To put it simply, the scaling coefficient is the slope of *F*(*l*) in a logarithmic presentation. An $\alpha$ between (0, 0.5) indicates a long-term anti-persistence characteristic, namely, the TS is anti-correlated. If this parameter takes values between 0.5 and 1, the TS is correlated, and its long-term persistence behavior can be studied. $1<\alpha <1.5$ implies a non-stationarity^57,58^. Besides, an $\alpha$ approximately equal to 1.5, 1 and 0.5 refers to Brownian, 1/*f* (pink), and white noise, respectively. The last corresponds to purely uncorrelated TS such as a random walk or an i.i.d. process^59,60^.

We performed the DFA on all the studied TS and verified their long-term correlations. The results are presented in “HVGs of SOC systems”.

## The classifier indicator

Applying the complex network approach, Lacasa et al.^61^ introduced the visibility graph (VG) method which converts a given TS into a graph. According to this algorithm, the sample data points are regarded as nodes (vertices), and an edge (link) is considered between nodes *i* and *j* if the following condition is met:

3 $$\begin{aligned} y_{c}<y_{i}+(y_{j}-y_{i})\frac{t_{c}-t_{i}}{t_{j}-t_{i}}, \end{aligned}$$

where $(t_{c}, y_{c})$ is a data value placed in between *i* and *j*. Further to this algorithm, Luque et al.^62^ presented the HVG which to a great extent is analogous to the VG method except for the visibility condition:

4 $$\begin{aligned} y_{i},y_{j} > y_{c} \quad (\text {for all c such that}~i< c < j). \end{aligned}$$

A major advantage of the HVG method is that it provides the analytical capability to calculate the degree distribution of random series. Therefore, the degree distribution of any random process regardless of the generator type follows a specific distribution:

5 $$\begin{aligned} P(k)=\frac{1}{3}{\bigg ( \frac{2}{3}\bigg )}^{k-2}, \end{aligned}$$

where *P* and *k* denote the probability distribution function (PDF) and the degree of nodes, respectively. The probability distribution of Eq. (5) could be regarded as an indicator for random series. In other words, if the degree distribution of a given sample follows Eq. (5) the underlying mechanism is most likely specified as a random process.

The question is how the degree distribution of other processes behaves in comparison with random TS. Luque et al.^62^ tried to address this question by performing a study on chaotic systems. They found that the degree distribution of chaotic processes **deviate** from the random indicator as they have higher probabilities at high degrees. Here, we go one step further and investigate the degree distribution of the total energy of SOC systems and examine their behavior against the random indicator. Moreover, we calculate the maximum eigenvalues and average clustering coefficients of several SOC systems and evaluate their utility in distinguishing between SOC and random systems. The details of the analysis and the achieved results are discussed in the next section.

## HVGs of SOC systems

The main idea of the present study is to investigate the SOC characteristics of data samples using the network approach. For this purpose, we construct the HVGs of various SOC systems and compare some of their networks’ properties (e.g., degree distribution) with random processes. But, we first need to validate the complexity of the subject TS.

In order to measure the possible deviations between the degree distributions of SOC and random TS, we calculate both the orthogonal regression and the Kolmogorov-Smirnov test (KS-test). Considering the random indicator as the reference, degrees with higher (lower) probabilities than the indicator are assigned with positive (negative) distances. Therefore, the sum of all distances specifies whether the subject system behaves randomly (approximate zero deviation), chaotically (positive deviation), or it represents SOC (negative deviation). Further to the degree distribution, we measure each network’s maximum eigenvalue which has been acknowledged as an efficient method to distinguish between chaotic and random TS^63,64^. Here, we appraise its capability in determining SOC systems. We also compute the average clustering coefficients^6,7,10,65,66^ of the HVGs and compare the outcomes with random processes.

We start our survey with the most celebrated SOC system, namely the sand-pile model^67^. This model employs a grid over which sand grains are initially randomly distributed. At each time step, a grain falls into a randomly selected square. The whole system is conned to a stability criterion that depends on the heights of nearest neighbors. Whenever a square does not contain enough capacity to accommodate the new grain (the height exceeds a pre-xed threshold), the grain ows into neighboring squares or ejects from the corners of the grid to locally relax the system.

**Figure 1:**
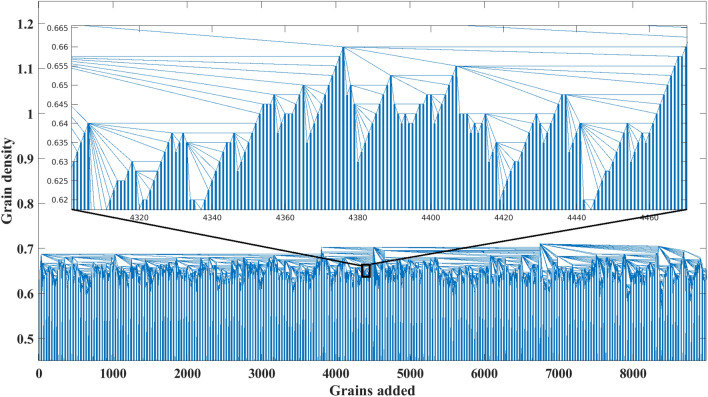
The HVG for the Manna sand-pile model with 9000 grains.

**Figure 2:**
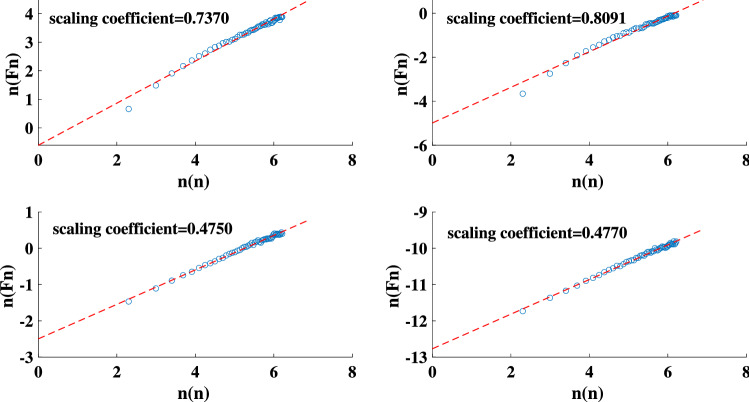
The scaling coefficient of the DFA for: the Bak sand-pile model (top left panel), Manna sand-pile model (top right panel), uniformly distributed random series (bottom left panel), and a normally distributed random series (bottom right panel).

We reproduce the sand-pile model using both the original Bak redistribution rules and those introduced by Manna (Fig. 1)^68^. The result of the performed DFA on both sand-pile models are presented in Fig. 2. The measured scaling coefficient of two random TS are also shown in the figure. According to the results, the scaling coefficients obtained with the DFA method for both Bak and Manna sand-pile models lies between 0.5 and 1. This indicates a long-term memory in these TS and confirms the complexity of the sand-pile systems.

**Figure 3:**
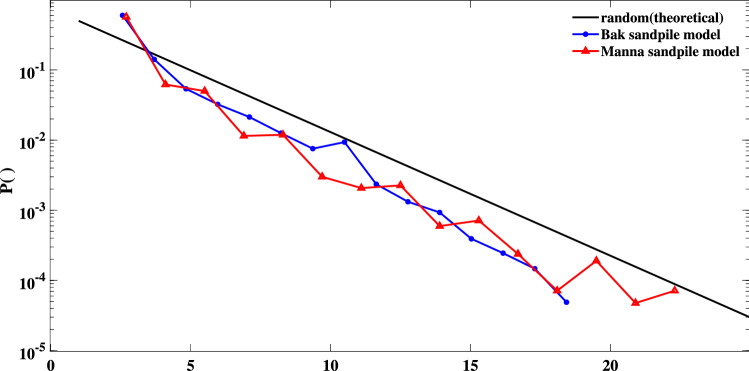
Semi-logarithmic presentation of degree distributions for: any random process (solid black line) which follows Eq. (5), the Bak (blue line), and Manna (red line) sand-pile models.

The degree distribution of both sand-pile models’ HVGs together with the theoretical distribution of random systems (Eq. 5) are shown in Fig. 3. The HVGs are constructed using the total energy of the SOC systems. As seen in the figure, most of the degrees (especially the high degrees) have less probabilities than the random indicator. The total deviation between the Bak and Manna sand-pile models and the indicator are $-5.9417$ and $-5.7680$, respectively. The orthogonal distances are measured in the logarithmic scale.

**Figure 4:**
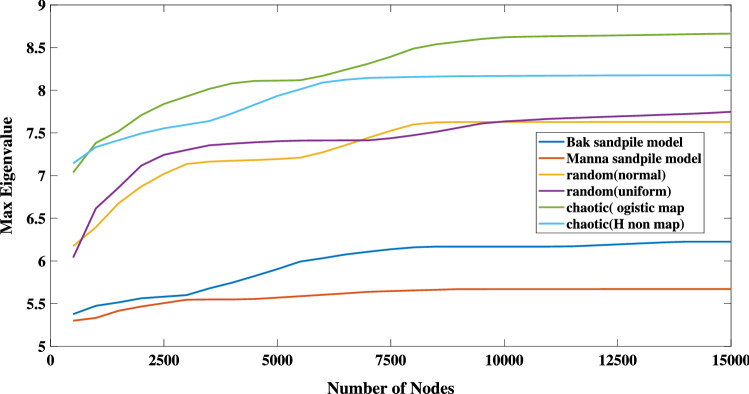
The maximum eigenvalues against the number of nodes for the constructed network for different TS.

**Figure 5:**
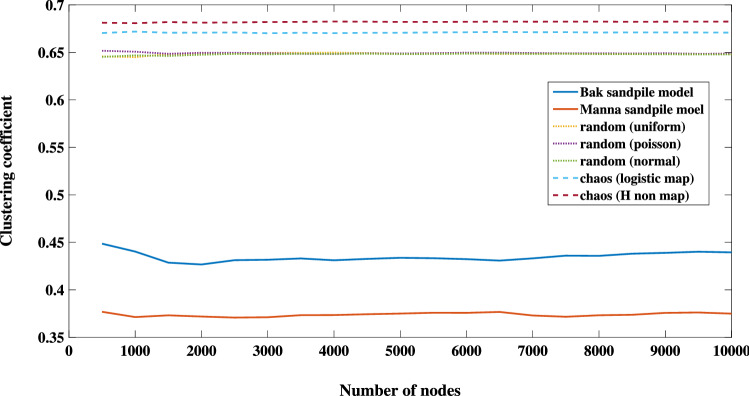
The average clustering coefficient for the Bak (solid blue line) and Manna (solid red line) models, several random TS (dotted colored lines), and two chaotic TS (dashed colored line).

Figure 4 displays the maximum eigenvalues of both sand-pile models together with the relevant values for some random and chaotic processes. The random TS are constructed using the uniform and normal generators whilst the chaotic TS correspond to a logistic map $x_{t+1}=\mu x_{t}(1-x_{t})$ and the Hénon map $(x_{t+1},y_{t+1})=(y_{t+1}-a x_{t}^{2},b x_{t})$ in a fully chaotic region with $\mu =4, a=1.4,$ and $b=0.3$. As shown in the figure, the maximum eigenvalues slightly increase with respect to the network’s size. For a convenient network with a total number of nodes greater than 2500, the maximum eigenvalues of a random and chaotic TS lie approximately in the ranges of 7–7.7 and 7.8–8.7, respectively. Whilst, the maximum eigenvalues of Bak and Manna sand-pile models are in the range of 5.5–6.3. The average clustering coefficients^6,7,10,65,66^ of mentioned TS are also presented in Fig. 5. Likewise, the maximum eigenvalues, and the average clustering coefficients of both sand-pile models are distinguishable from the random and chaotic processes.

So far, we have obtained that the HVGs of individual categories, random and SOC, seem to have distinctive characteristics. This raises the question of whether these properties (i.e., degree distribution, maximum eigenvalue, and average clustering coefficient) could practically be used as an indicator to identify SOC systems. To address this question, we examine several other SOC systems in the following.

Inspired by the power-law-like behavior of the solar flare energies, Tajfirouze and Safari^69^ presented a SOC model to investigate the complex evolution of nano-flare emissions in the quiet Sun and active regions. The nano-flare emission model is controlled by three free parameters, namely the power index $\alpha$, damping time $\tau _{d}$, and the occurrence probability of flaring events $p_{f}$^70–72^. The parameter $\alpha$ is the power index in the frequency-size distribution of flare energies (*E*):

6 $$\begin{aligned} \frac{dN}{dE} \propto E^{-\alpha }. \end{aligned}$$

**Figure 6:**
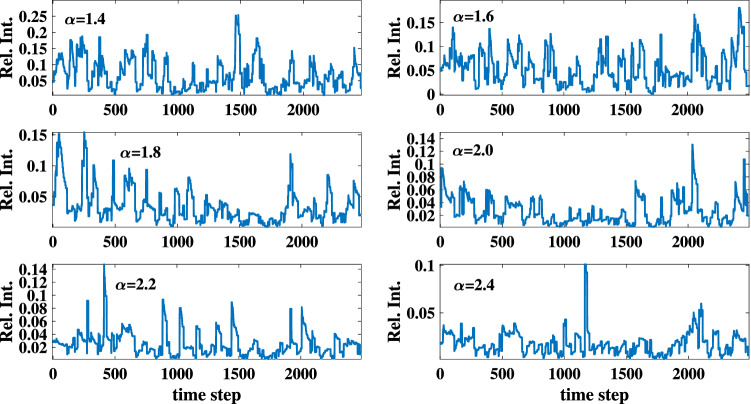
Simulated light curves of the nano-are emission model for $\tau _d=10$, $p_f =0.2$, and different $\alpha$ values.

The reported values for this parameter in solar and stellar flares lies between 1.5 and 2.9^23,24,70,72–81^. $\tau _{d}$ corresponds to the flare damping time and various choices of $\tau _{d}$ could affect the overall shape of the flares’ frequency-size distribution. For example, the adoption of large values for this parameter leads to a Gaussian frequency-size distribution rather than a power law. The parameter $p_{f}$ corresponds to the likelihood of a flare taking place and can take all values between 0 and 1. Figure 6 displays the simulated light curves of the nano-flare emission model for different sets of parameters ($\alpha \in$ [1.4 3.2], $\tau _{d} = 10,$ and $p_{f} < 0.2$).

**Figure 7:**
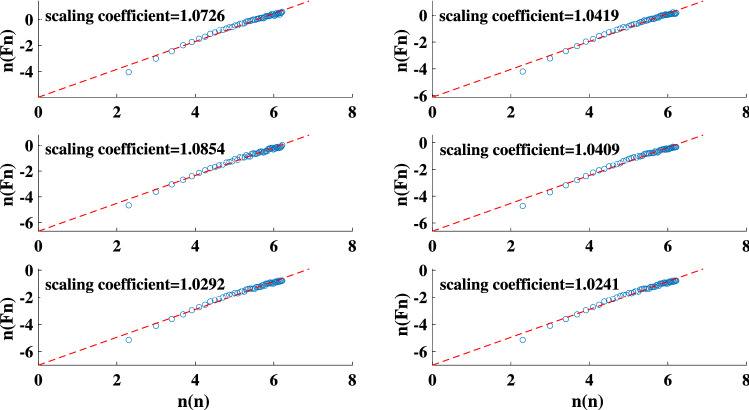
The scaling coefficient of the DFA for the simulated light curves of the nano-flare emission model with $\alpha$ =1.4, 1.6, 1.8, 2, 2.2, 2.4.

**Figure 8:**
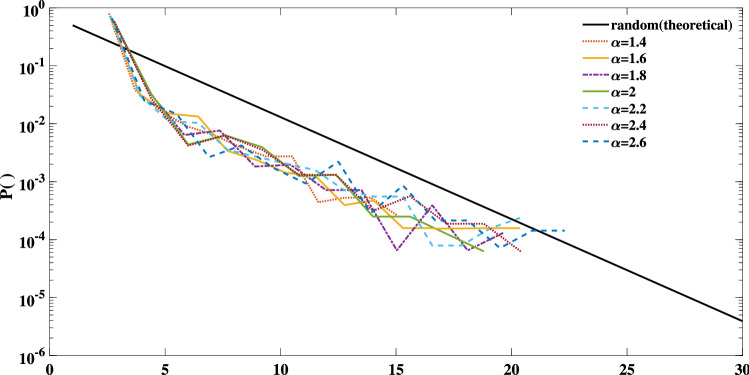
Semi-logarithmic presentation of degree distributions for: any random process (solid black line) which follows Eq. (5), and various runs of simulation of the nano-flare emission model with $\alpha \in$[1.4 3.2], $\tau _{d} = 10$, and $p_f =0.2$

Figures 7 and 8 present the result of the performed DFA and the degree distributions of the simulated light curves, respectively. The calculated scaling coefficients confirm the complexity of the simulated TS. All degree distributions exhibit a negative orthogonal regression from the random indicator. The deviation between each degree distribution and the indicator together with the maximum eigenvalues, and average clustering coefficients of each simulation are listed in Table 1. The results are compatible with the sand-pile models. The reported values are obtained by taking the average and standard deviations of each parameter for various sets of runs. Note that the procedure of repeating the calculations is not generally applicable to the real-world data sets except for conveniently large data samples.

**Table 1:** General properties of the constructed HVGs (with 14,000 nodes) for the nano-flare emission model.

$\alpha$	Orthogonal distances	Maximum eigenvalue	Clustering coefficient
1.4	– 11.3022	5.47 ± 0.16	0.27 ± 0.01
1.6	– 10.3042	5.64 ± 0.20	0.27 ± 0.01
1.8	– 7.9483	6.51 ± 0.21	0.28 ± 0.01
2	– 7.1418	6.03 ± 0.24	0.28 ± 0.01
2.2	– 6.9388	6.05 ± 0.16	0.27 ± 0.01
2.4	– 6.2146	6.38 ± 0.26	0.27 ± 0.01
2.6	– 5.0290	6.32 ± 0.39	0.23 ± 0.01

The model parameters are $\alpha \in$ [1.4 3.2], $\tau _{d} = 10,$ and $p_{f} < 0.2$.

**Figure 9:**
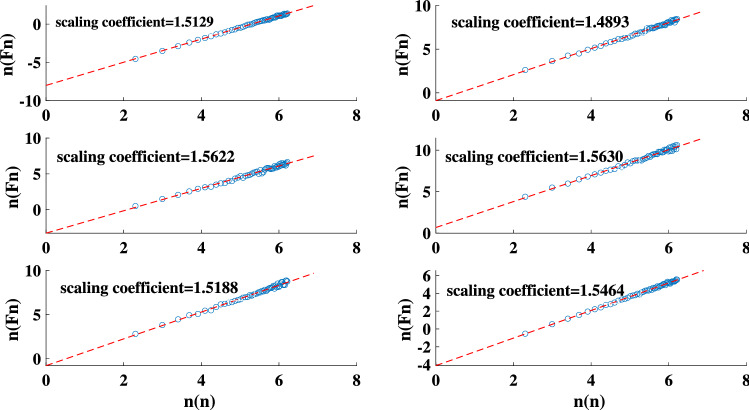
The scaling coefficient of the DFA for the exchange rate for euros to dollars (Euro/U.S. Dollar 1:1), gold price per ozt (Gold (ozt)/U.S. Dollar 1:1), Microsoft corp stock price, NASDAQ 100 index , *S* &*P* 500 index, U.S. dollar index.

**Figure 10:**
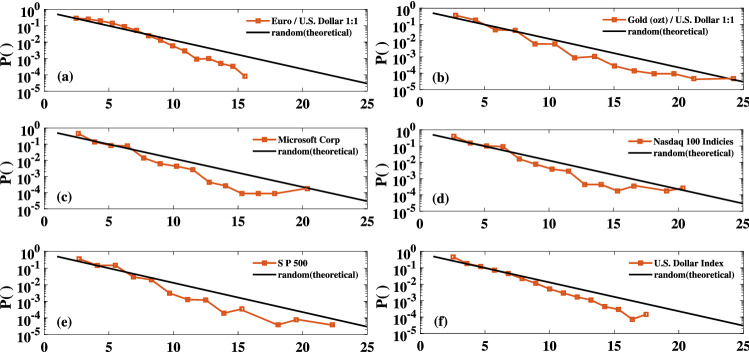
Semi-logarithmic presentation of PDFs of the degree of nodes for: any random process (solid black line) which follows Eq. (5), the exchange rate for euros to dollars (Euro/U.S. Dollar 1:1) from 04 January 1971 till 23 July 2021, gold price per ozt (Gold (ozt)/U.S. Dollar 1:1) from 01 March 1793 till 23 July 2021, Microsoft corp stock price from 13 March 1986 till 23 July 2021, NASDAQ 100 index from 1 October 1985 till 23 July 2021, S& P 500 index from 2 January 1952 till 23 July 2021, U.S. dollar index from 4 January 1971 till 23 July 2021.

We continue our survey by performing the same analysis on various financial data sets as other examples of SOC systems^22,82^. The dynamics of price movements or other indices of the financial markets are determined by the behavior of individuals who act based on their information^82^. Here, we investigate the historical price of several assets (such as gold, different company stocks, and commodities), as well as several economic indices (such as the Nasdaq 100, S &P 500, and U.S. dollar index). This information is registered in the Stooq Database and **is** online available at https://stooq.com/. We construct the HVGs for the maximum amounts of available data and the daily frequencies. Figure 9 exhibits the result of the DFA for the financial TS. The scaling coefficient of the subject series manifest the complex nature of these systems. Figure 10 displays the relevant degree distributions. The obtained orthogonal distances, maximum eigenvalues, and the average clustering coefficients of each HVG are presented in Table 2. Similar to previous SOC systems, the estimated parameters are less than the relevant values of random systems. However, the departures between the financial HVG’s clustering coefficients and the random systems are less significant compared to the sand-pile and nano-flare emission models.

**Table 2:** General properties of the financial TS HVGs.

Financial instrument	No. of nodes	Orthogonal distances	Maximum eigenvalue	Average clustering coefficient
Euro/U.S. Dollar	13070	$-$ 4.4967	5.7887	0.5875
Gold (ozt)/U.S. Dollar	14122	$-$ 4.9621	6.3913	0.5418
Microsoft Corp	9047	$-$ 6.4082	6.1790	0.5082
Nasdaq 100 Indicies	9162	$-$ 4.3530	6.3112	0.5922
S &P 500	19643	$-$ 5.3937	6.5304	0.5979
U.S. Dollar Index	13035	$-$ 5.0453	5.9480	0.5860

Further to the calculation of orthogonal distances, we apply the KS-test to compare the degree distribution of the HVGs with the random indicator (null hypothesis). The KS-test returns the test statistic (t-stat, the ratio of the departure between a specific model and the indicator to its standard deviation) and the p value (*p*)^83–85^. $p>0.1$ (threshold) rejects the null hypothesis and indicates that a degree distribution for a specific model may not obey the random indicator. Table 3 presents the obtained t-tests and p values for various random and SOC TS. As expected, the t-test values for both degree distributions of the normal and power-law random models satisfy the random indicator that are not rejected by p values ($p>0.1$). We observed that the degree distribution of the SOC TS deviates from the random indicator that **is** also rejected with the null hypothesis via the small p values ($p<0.1$).

**Table 3:** The result of the KS-test for all studied TS.

TS	t-test value	p values
Random power-law	0.0049	0.9998
Random normal	0.0041	0.9984
Bak	0.1358	$<0.0001$
Manna	0.2452	$<0.0001$
Chaos logistic map	0.1127	$<0.0001$
Chaos H$\acute{e}$non map	0.0777	$<0.0001$
$\alpha$ = 1.4	0.4546	$<0.0001$
$\alpha$ = 1.6	0.4513	$<0.0001$
$\alpha$ = 1.8	0.4482	$<0.0001$
$\alpha$ = 2	0.4497	$<0.0001$
$\alpha$ = 2.2	0.4506	$<0.0001$
$\alpha$ = 2.4	0.4480	$<0.0001$
$\alpha$ = 2.6	0.4457	$<0.0001$
Euro/U.S. Dollar	0.0621	$<0.0001$
Gold (ozt)/U.S. Dollar	0.0407	$<0.0001$
Microsoft Corp	0.0640	$<0.0001$
Nasdaq 100 Indicies	0.0617	$<0.0001$
S & P 500	0.0746	$<0.0001$
U.S. Dollar Index	0.0786	$<0.0001$

The two last columns of the table present the t-tests and p values for which the null hypothesis (random indicator) is accepted/rejected.

## Discussion

Determination of SOC has been always a challenge for the community. Although some evidence such as power-law frequency-size distribution of released energies usually inspires the idea of SOC, an extended knowledge of the underlying mechanism is required. In the present study, we argued the possibility of distinguishing SOC from random processes using their TS and appraised the capability of the complex network approach in this scheme. Particularly, we investigated the utility of the HVG in identifying the SOC characteristics of artificial and real-world data sets. We first constructed the HVGs of the Bak and Manna sand-pile models, the nano-flare emission model, and financial markets. Then, we validated the complexity of each TS by performing a DFA. Based on the obtained scaling coefficients, we confirmed that all studied TS differed from any uncorrelated random TS. We also computed the degree distribution, maximum eigenvalue, and *the* average clustering coefficient of each HVG and compared them with the relevant values for random processes.

**Figure 11:**
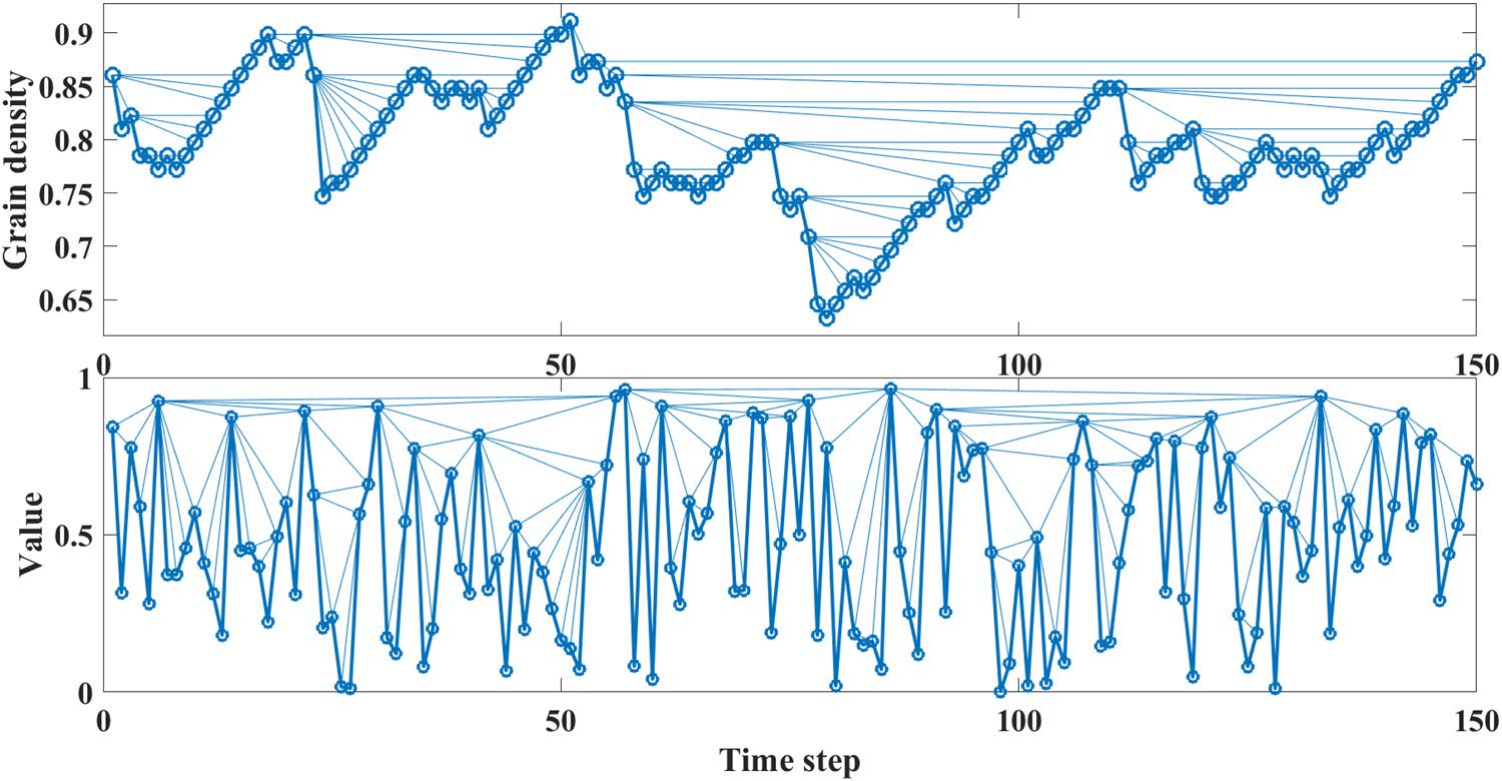
A HVG plot for a sample of 150 data points generated by the Manna sand-pile model (top panel), and a HVG of the same size TS generated by a uniform random algorithm (bottom panel).

Considering Eq. (5) as an indicator for any random process, we obtained the degree distributions of several SOC systems’ HVGs and evaluated their deviations from the random indicator. In all the studied TS, we found a negative orthogonal distance between the degree distribution of the subject system and the indicator (see e.g., Figs. 3 , 8, 10). In other words, we found that higher degrees are less probable in SOC systems rather than random processes. Therefore, an increase in the probability of high degrees indicates a transition from SOC to randomness or even chaos.

One may ask why the HVG’s degree distribution of a SOC TS lies below the random TS? To address this question, we compare a SOC TS generated via an avalanche mechanism (Fig. 11) with a randomly generated one. The first fluctuates slowly and it is called the load and unload mechanism. More specifically, due to the fact that it takes several consecutive steps for the system to experience either a growth or decay in its values, the variations appear slowly in the TS. However, in a random TS, a faster fluctuation is observed as after each kick or an extensive value the system holds to a minimal value due to the true nature of any random process. In practice, the time that it takes for a SOC system to build-up (or release) its energy controls the connectivity of the HVG’s network as it prevents having nodes with extensive connectivity. As shown in Fig. 11, the probability of having nodes with smaller degrees (e.g., $k=2,3$) in a SOC system is far greater than in a random TS. This, which has occurred due to the underlying generative mechanism of SOC, consequently leads to lower probabilities for higher degrees as $sum P(k) =1$. In other words, the probability function $P(k)=1/3(2/3)^{k-2}$ which is a baseline for random TS lies below the degree distribution of SOC TS at $k=3$. Also in a random TS, having the central nodes (nodes with large values) after some small values can provide high connectivity (the tail of degree distribution).

Furthermore, we found that the HVG’s maximum eigenvalues of any random process with at least 2500 data samples lie in the range of 7–7.8. However, the maximum eigenvalues of all the studied SOC systems are between 5.5 and 6.5. Similar departures are found between the average clustering coefficients of SOC and random processes.

We conclude that the obtained differences between the HVGs’ properties (degree distribution, maximum eigenvalue, and average clustering coefficient) of SOC and random systems originate in their generative mechanism and the HVG might be a useful tool in identifying SOC systems.

## Data Availability

The data for sand-pile and nano-flare emission models that support the findings of this study are available from the authors upon request [bardia.kaki@znu.ac.ir and safari@znu.ac.ir]. The financial markets data that support the findings of this study are available from [https://stooq.com/].
